# Thromboembolic events, bleeding, and drug discontinuation in patients with atrial fibrillation on anticoagulation: a prospective hospital-based registry

**DOI:** 10.1186/s12872-016-0438-5

**Published:** 2016-12-09

**Authors:** Oliver Königsbrügge, Alexander Simon, Hans Domanovits, Ingrid Pabinger, Cihan Ay

**Affiliations:** 1 Department of Medicine I, Clinical Division of Hematology & Hemostaseology, Medical University of Vienna, Währinger Gürtel 18-20, A-1090 Vienna, Austria; 2 Department of Emergency Medicine, Medical University of Vienna, Vienna, Austria; 3 Department of Medicine, Thrombosis and Hemostasis Program, McAllister Heart Institute, University of North Carolina at Chapel Hill, Chapel Hill, NC USA

**Keywords:** Atrial fibrillation, Anticoagulation, Tertiary healthcare, Stroke, Hemorrhage, Medication persistence

## Abstract

**Background:**

The clinical practice of stroke prevention in atrial fibrillation (AF) with direct oral anticoagulants (DOACS) differs from anticoagulation in randomized trial patients. We investigated the risk of thromboembolism, bleeding, and drug discontinuation in a hospital-based real-world setting.

**Methods:**

All-comer patients with non-valvular AF were recruited into a registry at an academic tertiary care center. After informed consent, patients underwent a personal structured interview including medical history, past and current anticoagulation, and returned for follow-up after 6–12 months.

**Results:**

The registry comprised 282 patients (42% women, median age 71 years) with a median CHA2DS2-Vasc-Score of 4 (25. to 75. percentile 2.5–5), who were prospectively followed 285 days in median. At inclusion, 118 patients took vitamin-K-antagonists, 33 dabigatran, 87 rivaroxaban, 30 apixaban, 5 low-molecular-weight heparin, and 9 were on no anticoagulant. Occurrence of stroke (rate 2.8/100 patient-years), was associated with prior stroke (hazard ratio [HR] 18.5, 95% confidence interval 2.16–159), increased HbA1c (HR per 1% increase 1.71, 1.20–2.45) and borderline significantly associated with vascular disease (HR 8.33, 0.97–71.3). Further we observed a high rate of major bleeding (2.8/100 patient-years), clinically relevant non-major bleeding (4.1/100 patient-years), and venous thromboembolism (2.8/100 patient-years). Anticoagulation was discontinued by 80 patients (36.9/100 patient-years), and diabetes (HR 2.31, 1.32–4.02), history of bleeding (HR 2.51, 1.44–4.37) and elevated leucocyte count (HR per 1G/l increase 1.02, 1.00–1.05) were associated with increased risk of discontinuation.

**Conclusions:**

In this hospital-based registry, patients with atrial fibrillation had an increased risk of thromboembolic events despite anticoagulation. The low drug persistence may be attributable to distinct comorbid conditions and bleeding complications.

## Background

The increased risk of stroke and systemic embolism in patients with non-valvular atrial fibrillation (AF) can be attenuated with continuous oral anticoagulation treatment [1]. Direct oral anticoagulants (DOACs), including the direct thrombin inhibitor dabigatran and the direct factor Xa inhibitors rivaroxaban, apixaban, and edoxaban, have shifted the paradigm of anticoagulation treatment from routine drug monitoring and dose adjustment to a one-size-fits-all strategy, and changed clinical practice of oral anticoagulation. Real-world data have confirmed the efficacy and safety of DOACs for stroke prevention [2, 3]. However, in a hospital-based patient population with AF, comorbid conditions, comedications and surgical interventions may complicate treatment with anticoagulant drugs, which has not been specifically addressed in previous real-world investigation.

Furthermore, there is still concern that the ease of drug administration and clinical management with DOACs may not guarantee better persistence on treatment in a real-world setting. Drug persistence is defined as the total time a patient stays on a prescribed medication and reduced persistence increases the risk of stroke [4, 5]. In the randomized controlled trials that led to the licensing of DOACs for stroke prevention in AF, 20.7%–34.3% of patients receiving DOACs discontinued drug treatment during the study period and 16.6%–34.4% discontinued the control treatment, warfarin [6–9]. However, drug persistence in clinical trials may be completely different from real-world persistence, because there are different incentives for remaining on treatment in real-world patients.

We aimed to examine the characteristics of non-valvular AF patients in a hospital-based setting and investigate risk factors for occurrence of thromboembolic events, bleeding episodes and drug persistence while on anticoagulant treatment, as well as reasons for drug discontinuation.

## Methods

### Patients

Patients with non-valvular AF referred to the Clinical Division of Hematology and Hemostaseology were recruited into a hospital-based, prospective registry from July 2013 to May 2016. Inclusion criteria were confirmed diagnosis of AF by a 12-channel, resting electrocardiogram (ECG), willingness to comply with study procedures, and written informed consent. The study has approval of the local ethics committee. There were no exclusion or selection criteria concerning anticoagulation treatment, medical history, risk of stroke, or bleeding. After obtaining informed consent for study participation, a study investigator performed a personal structured interview with each patient, recorded the medical history and a detailed anticoagulation history. Patients returned for voluntary, scheduled follow-up visits 6 and 12 months after recruitment.

### Baseline assessment of patient characteristics

All baseline patient data was recorded from personal interviews and verified against medical records. The structured interview included a detailed medical history, especially concerning prior thromboembolic events, and a detailed record of previous periods of anticoagulation treatment and associated complications. During baseline data collection, a history of vascular disease was defined as arterial disease of any degree including coronary heart disease, peripheral artery disease, and carotid artery disease. Cancer was defined as any history of malignancy or active malignancy at study inclusion with the exception of basalioma. Patients were anticoagulation naive at baseline if they had not received continuous anticoagulation treatment previously for longer than 3 months. The baseline kidney function was calculated on the grounds of the estimated glomerular filtration rate (eGFR) and isotope-dilution mass spectrometry (IDMS) traceable serum creatinine levels using the Modification of Diet in Renal Disease (MDRD) equation. The risk of stroke was assessed with the CHA_2_DS_2_-Vasc score and the risk of bleeding with the HAS-BLED score.

### Definition of events during prospective observation

Bleeding outcomes were classified according to the recommendations of the Scientific and Standardization Committee of the International Society of Thrombosis and Hemostasis. Major bleeding was defined as clinically overt bleeding with fatal outcome, involvement of a critical anatomic site, fall in hemoglobin concentration of more than 2 g/dl, transfusion of >2 units of whole blood or packed red blood cells, or leading to permanent disability [10]. Clinically relevant non-major (CRNM) bleeding was defined as overt bleeding not meeting criteria for major bleeding but requiring medical intervention, hospitalization, temporary interruption or delayed dosing of anticoagulation, pain, or impairment of daily activities [11]. Minor bleeding was defined as any other bleeding not meeting the above criteria. Venous thromboembolism (VTE) was defined as objectively confirmed deep vein thrombosis (DVT) or pulmonary embolism (PE). Stroke was defined as the sudden onset of a distinct focal neurologic deficit in a location consistent with the territory of a major cerebral artery and conclusive imaging evidence. In the absence of imaging evidence, but conclusive neurological symptoms confirmed by a specialist in neurology, we recorded a transient ischemic attack (TIA). All events were internally validated according to the criteria listed above.

### Definition of anticoagulation persistence

Drug persistence was defined as the time in weeks from the initiation of anticoagulation until discontinuation of treatment [12]. Only patients who initiated anticoagulation at the time of recruitment were included in the persistence analysis. The percentage of patients remaining on the baseline anticoagulant was calculated for 6 and 12 months as well as the annualized discontinuation-rate. The end of anticoagulation persistence was defined as the permanent cessation of anticoagulation treatment or switching from one anticoagulant to a different anticoagulant, but did not include pausing anticoagulation treatment for a limited time of less than 1 month.

### Statistical methods

Baseline patient characteristics of the registry cohort were described as absolute and relative frequencies or with median and 25^th^ to 75^th^ percentile, respectively for categorical and continuous data. The baseline group comparisons between DOAC and vitamin-K-antagonist (VKA) patients were calculated with the Mann–Whitney-U test for categorical parameters or the chi^2^ test for continuous variables. An asymptotic two-sided *p*-value of smaller than 0.05 was considered statistically significant. The risks of stroke, TIA, or systemic embolism, bleeding, and drug discontinuation were calculated with the univariable Cox proportional hazards model. All calculations were performed with SPSS (Windows Version 23.0; IBM Corp., Armonk, NY, http://www.ibm.com). Risk of discontinuation was only calculated with patients who initiated a new anticoagulation therapy at study inclusion.

## Results

### Baseline registry characteristics

The registry includes 282 patients (118 [42%] women, 164 [58%] men) with ECG-confirmed diagnosis of AF and a median age of 71 years (25^th^ to 75^th^ percentile 65 – 77 years). At baseline, 118 patients (41.8%) were on VKA, 33 patients (11.7%) on dabigatran, 87 (30.9%) on rivaroxaban, 30 (10.6%) on apixaban, 5 patients (1.8%) on long-term low-molecular-weight heparin (LMWH) and 9 patients (3.2%) did not take any anticoagulant drug. The baseline characteristics of the full registry and respective to groups of patients on each anticoagulant are shown in Table 1. DOACs were more frequently prescribed to patients with more recently diagnosed AF and to patients previously naïve to anticoagulation treatment (Table 1). Patients with history of CRNM or major bleeding, with age between 65–75 years were also more frequently on DOAC treatment. Patients older than 75 years were more often on VKA, while patients on DOACs, especially rivaroxaban and apixaban, tended to have lower hemoglobin and hematocrit levels than patients on VKA. Patients with lower eGFR, however, tended to take VKA (Table 1).

**Table 1:** Baseline cohort characteristics

Characteristic	Full cohort (*N* = 282)	VKA (*N* = 118)	Dabigatran (*N* = 33)		Rivaroxaban (*N* = 87)		Apixaban (*N* = 30)	
				p*		p*		p*
Age	71 (65–77)	73 (67–79)	73 (68–77)	0.8	71 (66–75)	0.1	71 (64–75)	**0.04**
Female sex	116 (43.1)	47 (41.6)	11 (34.4)	0.5	30 (37.0)	0.5	22 (75.9)	**0.001**
BMI	27.0 (24.5–30.4)	27.3 (24.9–31.2)	27.3 (25.0–31.0)	0.7	27.0 (24.0–31.1)	0.5	26.9 (22.2–28.3)	0.09
Type of AF								
Recently diagnosed	52 (18.4)	10 (8.5)	8 (24.2)	**0.04**	16 (18.4)	0.2	13 (43.3)	**<0.001**
paroxysmal	131 (46.5)	60 (50.8)	10 (30.3)	**0.05**	42 (49.4)	0.8	12 (40.0)	0.3
persisting	19 (6.7)	9 (7.6)	1 (3.0)	0.4	7 (8.6)	0.7	1 (3.3)	0.5
permanent	80 (28.4)	39 (33.1)	14 (42.4)	0.3	22 (27.2)	0.4	4 (13.3)	**0.04**
Time since AF diagnosis, years	4 (1–8)	4 (2–9)	5 (0.1–9)	0.5	3 (1–10)	0.1	2 (0–6)	**0.01**
History of electrical cardioversion	83 (30.9)	40 (35.4)	10 (31.3)	0.7	21 (25.9)	0.2	9 (31.0)	0.7
History of ablation	35 (13.0)	15 (13.3)	5 (15.6)	0.7	13 (16.0)	0.6	1 (3.4)	0.1
Family history of AF	33 (12.3)	13 (11.5)	3 (9.4)	0.7	10 (12.3)	0.9	7 (24.1)	0.08
Medical history								
Congestive heart failure	81 (30.1)	34 (30.1)	13 (40.6)	0.3	24 (29.6)	0.9	8 (27.6)	0.8
Hypertension	227 (84.4)	99 (87.6)	30 (93.8)	0.3	65 (80.2)	0.2	26 (89.7)	0.8
Age ≥ 75 years	98 (36.4)	53 (46.9)	13 (40.6)	0.5	23 (28.4)	**0.009**	8 (27.6)	0.06
Age 65–74 years	114 (42.4)	41 (36.3)	14 (43.8)	0.4	43 (53.1)	0.02	13 (44.8)	0.4
Diabetes	81 (28.7)	31 (27.4)	10 (31.3)	0.4	22 (27.2)	0.7	8 (27.6)	0.9
Stroke / TIA / systemic embolism	55 (20.4)	22 (19.5)	7 (21.9)	0.8	20 (24.7)	0.4	4 (13.8)	0.5
Vascular disease	96 (35.7)	41 (36.3)	13 (40.6)	0.7	28 (34.6)	0.8	11 (37.6)	0.9
CHA_2_DS_2_-VASC Score	4 (2.5–5)	4 (3–5)	4 (3–5)	0.9	3 (2–5)	0.3	4 (3–4.5)	0.4
HAS-BLED Score	2 (1–3)	2 (1–2)	2 (1–3)	0.7	2 (1–3)	0.2	2 (1–3)	0.5
Abnormal liver or kidney function	52 (19.3)	24 (21.2)	5 (15.6)	0.5	18 (22.2)	0.9	5 (17.2)	0.6
CRNM bleeding	13 (4.8)	1 (0.9)	2 (6.3)	0.06	4 (4.9)	0.08	4 (13.8)	**0.001**
Major bleeding	10 (3.7)	1 (0.9)	5 (15.6)	**<0.001**	4 (4.9)	0.08	0	0.6
Venous thromboembolism	22 (8.2)	7 (6.2)	3 (9.4)	0.5	9 (11.1)	0.2	3 (10.3)	0.4
Cancer	58 (21.6)	19 (16.8)	9 (28.1)	0.2	19 (23.5)	0.3	6 (20.7)	0.6
OAC-naïve at baseline	18 (6.7)	4 (3.5)	2 (6.3)	0.5	6 (7.4)	0.2	4 (13.8)	**0.03**
Aspirin comedication	58 (21.6)	23 (20.4)	5 (15.6)	0.6	19 (23.5)	0.6	6 (20.7)	0.9
Clopidogrel comedication	14 (5.2)	9 (8.0)	2 (6.3)	0.7	3 (3.7)	0.2	0	0.1
Baseline laboratory parameters								
Platelet count	214 (176–264)	211 (180–251)	203 (172–239)	0.4	215 (174–286)	0.3	238 (177–275)	0.4
Hemoglobin	13.2 (11.8–14.4)	13.5 (12.5–14.8)	13.2 (11.8–15.2)	0.3	12.7 (11.4–14.1)	**0.002**	12.8 (11.0–14.1)	**0.05**
Hematocrit	39.5 (35.1–42.8)	40.3 (37.4–43.9)	39.5 (34.9–42.6)	0.2	38.5 (33.6–41.0)	**0.001**	38.8 (33.9–41.2)	**0.03**
Leucocyte count	6.8 (5.7–8.5)	6.8 (5.7–7.9)	7.0 (6.0–8.6)	0.3	6.9 (5.7–8.9)	0.3	6.4 (5.2–7.9)	0.3
eGFR (ml/min/1.73 m^2^)	65.2 (53.3–79.6)	60.4 (49.5–74.3)	71.2 (58.4–91.8)	**0.03**	66.8 (56.2–82.1)	**0.01**	65.8 (56.6–73.1)	0.2

Footnote: * Mann–Whitney-U test or chi^2^ *p*-value for asymptomatic two-sided difference between respective DOAC group and VKA group, statistically significant *p*-values in bold print

*Abbreviations*: *AF* atrial fibrillation, *VKA* Vitamin-K-antagonist, *BMI* body-mass-index, *TIA* transient ischemic attack, *CRNM bleeding* clinically relevant non-major bleeding, *OAC* oral anticoagulation, *eGFR* estimated glomerular filtration rate

### Prospective outcomes

Prospective follow-up was available for 269 patients and 13 patients (4.6%) were lost to follow-up. The median observation time was 285 days (227–405 days) (minimum 1 day, maximum 966) for a total of 217 patient-years of observation time. During follow-up, 6 (2.2%) cardioembolic events occurred (4 ischemic strokes, 1 TIA, 1 systemic embolism), corresponding to an event-rate of 2.8 per 100 patient-years. Of these events, 4 occurred while on VKA, 1 while on rivaroxaban, and 1 while on triple therapy with VKA.

In univariable Cox regression, patients with a history of stroke, TIA, or systemic embolism had an 18-fold increased risk of a new stroke, TIA or systemic embolism (hazard ratio [HR] 18.5, 95% confidence interval [CI] 2.16–159, *p* = 0.008), patients with vascular disease had a borderline significant 8.3-fold increased risk of stroke (HR 8.3, 95% CI 0.97–71.3, *p* = 0.05), and for every 1% increase in HbA1c the risk of stroke increased 1.7-fold (95% CI 1.20–2.45, *p* = 0.003) (Table 2). For every one point added to the CHA2DS2-Vasc score the risk of stroke doubled (HR 2.06, 95% CI 1.27–3.35, *p* = 0.004). One myocardial infarction occurred while on VKA, and 6 VTE events occurred (4 DVT, 2 PE), corresponding to an event-rate of 2.8 per 100 patient-years. One VTE event occurred while on rivaroxaban therapy, 2 during rivaroxaban pause, 1 during VKA treatment, 1 during VKA pause, and 1 after discontinuation of rivaroxaban. Major bleeding events occurred in 6 patients (3 intraocular hemorrhages, 2 intraabdominal bleedings with massive blood loss and/or transfusions, 1 subdural hematoma) and CRNM bleeding events occurred in 9 patients (5 gastrointestinal [GI] bleeds, 4 other bleeds requiring hospitalization). The event rate for major bleeding was 2.8 per 100 patient-years and for CRNM bleeding 4.1 per 100 patient-years. Minor bleeding events occurred in 43 patients and 15 patients died during follow-up of causes unrelated to atrial fibrillation or anticoagulation. The results of our analysis revealed no statistically significant risk factor for bleeding that occurred during follow-up.

**Table 2:** Univariable Cox regression analysis of risk factors for the outcomes stroke, bleeding, and drug discontinuation

	Hazard of stroke, TIA, systemic embolism (*N* = 269)		Hazard of CRNM or major bleed (*N* = 269)		Hazard of anticoagulant discontinuation (*N* = 144)	
Characteristic	Hazard ratio (95% confidence interval)	*p*-value	Hazard ratio (95% confidence interval)	*p*-value	Hazard ratio (95% confidence interval)	*p*-value
Age^a^	0.98 (0.90–1.06)	0.59	1.03 (0.97–1.09)	0.30	0.99 (0.96–1.02)	0.54
Female sex	6.45 (0.75–55.2)	0.09	0.95 (0.36–2.56)	0.93	1.01 (0.59–1.75)	0.96
BMI^a^	0.96 (0.81–1.13)	0.63	1.00 (0.92–1.10)	0.96	0.99 (0.94–1.04)	0.65
Medical history						
Congestive heart failure	2.00 (0.40–9.94)	0.40	1.15 (0.42–3.18)	0.78	1.28 (0.73–2.23)	0.39
Hypertension	24.2 (0.00–999)	0.60	23.8 (0.01–999)	0.42	0.60 (0.28–1.28)	0.18
Diabetes	2.42 (0.49–12.0)	0.28	1.13 (0.39–3.27)	0.82	2.31 (1.32–4.02)	**0.003**
Stroke/TIA/systemic embolism	18.5 (2.16–159)	**0.008**	0.76 (0.22–2.69)	0.67	0.76 (0.38–1.53)	0.44
Vascular disease	8.33 (0.97–71.3)	0.05	0.62 (0.21–1.82)	0.39	1.16 (0.67–2.00)	0.61
CHA_2_DS_2_–VASC Score^a^	2.06 (1.27–3.35)	**0.004**	1.03 (0.77–1.37)	0.85	1.05 (0.90–1.21)	0.57
History of bleeding	0.85 (0.10–7.27)	0.88	1.17 (0.33–4.15)	0.81	2.51 (1.44–4.37)	**0.001**
HAS-BLED Score^a^	1.61 (0.90–2.87)	0.11	1.18 (0.77–1.80)	0.45	1.14 (0.92–1.42)	0.22
Venous thromboembolism	2.26 (0.26–19.4)	0.46	0.87 (0.11–6.64)	0.89	1.29 (0.51–3.26)	0.59
Active cancer/history of cancer	0.04 (0.00–330)	0.48	1.24 (0.35–4.40)	0.74	1.15 (0.62–2.12)	0.66
OAC-naïve at baseline	0.05 (0.00–999)	0.69	0.94 (0.12–7.23)	0.95	0.78 (0.31–1.97)	0.60
Baseline laboratory parameters (hazard ratios per 1 unit increase)						
Platelet count^a^	1.00 (0.99–1.01)	0.71	1.00 (0.99–1.01)	0.60	1.00 (0.99–1.00)	0.34
Hemoglobin^a^	0.80 (0.52–1.22)	0.29	1.05 (0.81–1.37)	0.71	0.89 (0.79–1.02)	0.08
Hematocrit^a^	0.95 (0.81–1.11)	0.51	1.01 (0.91–1.11)	0.86	0.96 (0.92–1.01)	0.11
Leucocyte count^a^	1.02 (0.91–1.13)	0.77	0.82 (0.63–1.06)	0.12	1.02 (1.00–1.05)	**0.041**
eGFR^a^	0.97 (0.93–1.01)	0.17	0.98 (0.96–1.01)	0.21	1.01 (1.00–1.02)	0.27
HbA1c^a^	1.71 (1.20–2.45)	**0.003**	1.13 (0.75–1.72)	0.54	1.31 (0.87–1.98)	0.20

Legend: *BMI* body-mass index, *TIA* transient ischemic attack, *VKA* vitamin-K-antagonist, *OAC* oral anticoagulant, *eGFR* estimated glomerular filtration rate, 999 as the upper bound of the 95% confidence interval signifies an abbreviation of a very wide confidence interval, *p*-values in bold font represent statistically significant findings, ^a^ the hazard ratios for continuous variables are given as per 1 unit increase: age in years, BMI in kg/m^2^, platelet count in G/l, hemoglobin in g/dl, hematocrit in %, leucocyte count in G/l, eGFR in ml/min/1.73 m^2^, HbA1c in rel.%, and D-dimer in μg/ml

### Anticoagulation persistence

**Fig. 1:**
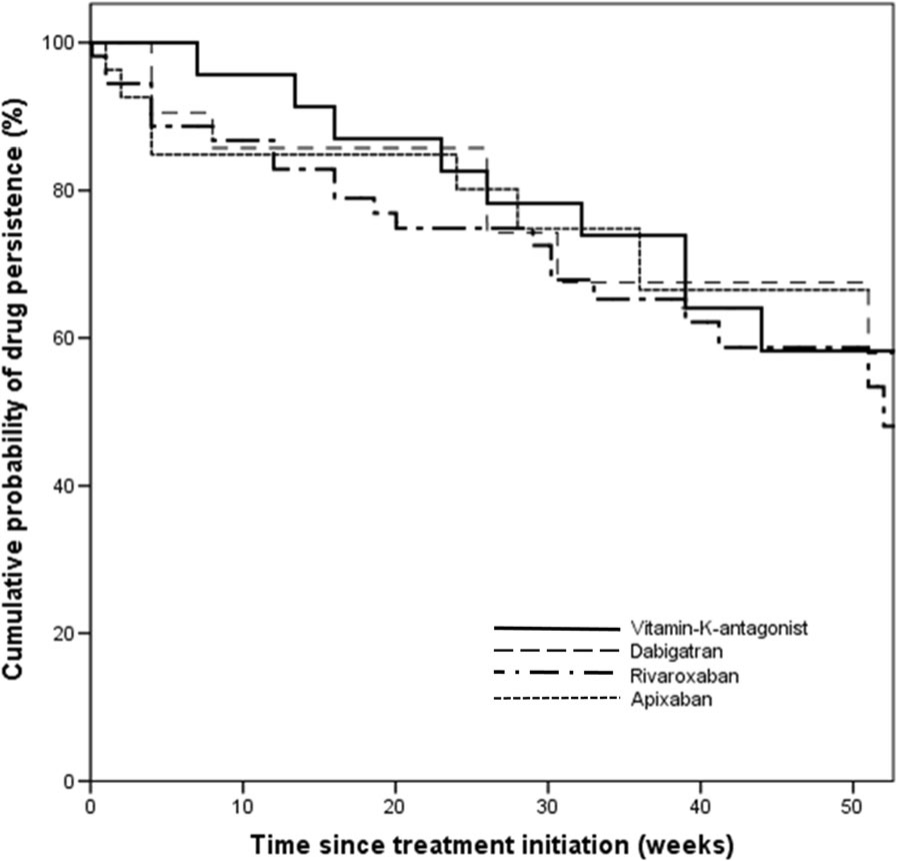
Cumulative drug persistence over the course of 50 weeks

The median persistence was 32 weeks (25^th^ to 75^th^ percentile: 12 to 46 weeks) and 80 patients (29.7%) discontinued the anticoagulation therapy, which they had received at baseline. This corresponds to a rate of discontinuation of 36.9 per 100 patient-years. After 6 months, the overall drug persistence of the baseline anticoagulant was 76.7% and after 12 months further reduced to 54.7%. There was no difference in the persistence between patients receiving DOACs and VKA (Fig. 1). The most frequent reasons for discontinuing anticoagulation were patient-reported end of AF and permanent return to sinus rhythm (20%), emergence of a new contraindication for current anticoagulation treatment (15%) (e.g. mechanical heart valve), physician’s recommendation (12.5%), and occurrence of major or CRNM bleeding events (11.3%) (Table 3). The choice for an alternative anticoagulant after discontinuation of the baseline anticoagulant drug was evenly distributed between VKA, rivaroxaban, apixaban or no anticoagulant at all (Table 3). In regression analysis, patients with diabetes had a 2.3-fold increased risk of discontinuation (95% CI 1.32 to 4.02), patients with history of bleeding had a 2.5-fold increased risk (95% CI 1.44 to 4.37) and per 1 G/l increase in leucocyte count the risk of discontinuation increased by 2% (HR = 1.02, 95% CI 1.00 to 1.05).

**Table 3:** Reasons for discontinuation of first choice anticoagulant and frequency of alternative choice anticoagulants (N = 80)

	Frequency (%)
Reason for discontinuation	
Patient-reported permanent return to sinus rhythm	16 (20.0)
Contraindication	12 (15.0)
Physician’s recommendation	10 (12.5)
Difficulty reaching INR 2–3 (VKA only)	9 (11.3)
Major or clinically-relevant non-major bleed	9 (11.3)
Thromboembolism	6 (7.5)
Minor bleeding	5 (6.3)
Renal insufficiency	3 (1.1)
Patient´s wish	3 (1.1)
other	7 (8.8)
Alternative choice anticoagulant	
Vitamin-K-Antagonist	16 (20.0)
Dabigatran	8 (10.0)
Rivaroxaban	16 (20.0)
Apixaban	18 (22.5)
LMWH (long-term)	5 (6.3)
None	17 (21.3)

## Discussion

In this analysis of a real-world, tertiary-care, hospital-based registry of patients with atrial fibrillation, the rate of cardioembolic events (2.8 per 100 patient-years) was higher than in the randomized controlled trials for stroke prevention in AF. In RE-LY, ROCKET-AF, ARISTOTLE and ENGAGE-AF-TIMI48, 1.6–2.2% of patients randomized to warfarin and 1.11–2.04% for patients randomized to the study drugs had strokes or systemic embolisms per year [6–9]. The stroke-rate in our study was also higher than in other data sets of real-world AF patients. Graham et al. reported a stroke rate of 1.1 per 100 patient years for dabigatran and 1.4 for warfarin in a database of US Medicare patients [13]. Korenstra et al. found a rate of stroke of 0.8% per year for VKA and 1.0% per year for dabigatran outpatients [14], and Hecker et al. reported an event rate of 2.0 per 100 patient-years in rivaroxaban patients in daily-care [15]. In a recent report from the GARFIELD-AF registry, the rate of stroke was 1.25 per 100 patient-years [16]. Not surprisingly, these cohorts had lower frequencies of risk factors for strokes, including a prior history of stroke, a history of vascular disease, diabetes and a decreased CHA2DS2-Vasc Score, compared to our cohort. This is confirmation that a stroke risk evaluation is clinically meaningful in a hospital-based setting. We also observed a rate of myocardial infarction of 0.5 per 100 patients-years and a rate of VTE of 2.8 per 100 patient-years. Firstly, it is interesting that a cohort reported by Beyer-Westendorf et al. from a population of primary-care AF patients with similar baseline stroke risk factors and similar stroke rate had no VTE events at all [2]. Thus, the risk of VTE in AF patients, especially when medically ill or frail, should not be neglected. Secondly, a closer inspection of the circumstances under which VTE events occurred, revealed that the majority occurred during temporary pausing of the anticoagulation therapy. Thus, VTE events may have been avoidable with a proper bridging strategy or minimizing of the anticoagulation pause. We would like to interpret this signal as a call to action on emphasizing educational programs on safe and efficacious anticoagulation management during interventions, operations, and hospitalizations.

Major bleeding events occurred with a rate of 2.8 per 100 patient-years and CRNM bleeding events with a rate of 4.1 per 100 patient-years. The rate of major or CRNM bleeding events was, however, not elevated in our cohort compared to some other real-world registries despite a median age of 71 years and a history of severe bleeding complications in 8.5% of patients. This may be an indication that bleeding risk evaluation in medically ill patients may be different from the general population and that bleeding assessment tools, such as the HAS-BLED score, should be updated to the DOAC era. Three patients, without diabetes or previous history of intraocular or retinal bleeding, suffered from symptomatic, intraocular hemorrhage during follow-up. Bleeding in this atypical site has previously been reported in trials on anticoagulation in AF patients [7], but the high frequency in this cohort is nonetheless surprising and may warrant more awareness among physician who treat patients with anticoagulation.

We further investigated persistence on anticoagulation treatment in this registry and found that 80 patients (29.7%) discontinued their baseline anticoagulation over the course of the follow-up, which translates to a discontinuation rate of 36.9 per 100 patient-years. The majority, 69 of them, switched to a different anticoagulant agent, but 11 patients discontinued anticoagulation treatment altogether. We found that after 6 months the drug persistence on the baseline anticoagulant agent was 76.7% and after 12 months further reduced to 54.7%. The overall persistence in the registry is lower than in some previous reports. While in the pre-DOAC era, discontinuation frequency had been 26–28% [17, 18], Beyer-Westendorf et al. reported a persistence rate of 81.5% in patients on treatment with rivaroxaban after a median treatment time of 544 days [19]. The most frequent reason for discontinuation of anticoagulation treatment in our registry was permanent return to sinus rhythm. This group of patients is perceived as a low-risk group and especially after successful ablation treatment, anticoagulation treatment is oftentimes discontinued in clinical practice [20]. There is, however, no evidence supporting a discontinuation of anticoagulation in patients reporting a permanent return to sinus rhythm, because AF may be asymptomatic in nature.

Moreover, we identified novel patient characteristics that decrease anticoagulation persistence. A history of bleeding, although not a risk factor for occurrence of major or CRNM bleeding was a strong risk factor for discontinuation and the composite of all bleeding events was the second most frequent reason for anticoagulation discontinuation. Further, patients with diabetes and increased leucocyte count had increased risk of discontinuation. These patients had to discontinue their baseline anticoagulation treatment predominately for emerging chronic medical issues, such as deterioration of kidney function, coronary heart and peripheral artery disease, and for safety considerations because of initiation of antiplatelet medication.

Although our registry was not intended for a direct comparison of the persistence between different anticoagulants, our data allow a cautious analysis of drug persistence on VKA and on DOACs. Patients receiving DOACs were younger, had a higher frequency of previous bleeding and had more frequently a recent diagnosis of AF. Nevertheless, patients receiving DOACs did not have inferior drug persistence compared to patients receiving VKA. In studies in German and UK primary care settings, patients on treatment with DOACs even had better drug persistence than patients on VKA [21, 22]. The benefit of DOACs over VKA concerning persistence may have been attenuated in the hospital setting, because treating physicians were more cautious in the use of DOACs when new medical complications emerged.

This present investigation was not without limitations. Patients were not randomized to anticoagulation treatment, which may have led to a selection bias in the distribution of comorbidities between DOAC patients and VKA patients. For example, those patients who remained on VKA treatment during the observation time were likely long-time VKA users with a stable management of the international-normalized ratio (INR) and personal positive experience with VKA use. The study was not suitable for assessing time in therapeutic range because INR values in between follow-up visits were not captured. Analysis of safety or efficacy between drug classes were also not permissible because there was no randomization to anticoagulation treatment. Patients were referred to the outpatient clinic for the purposes of study inclusion and not based on any selection criteria such as high risk of bleeding or stroke. There may however be an inherent selection bias in the small sample size. The low sample size further led to wide confidence interval in the logistic regression analysis. Members of the registry staff did not actively prescribe medications or suggest drug discontinuation, nor did they influence the choice of DOAC. However, in cases of adverse events, patients were encouraged to seek the advice of a physician who is independent from the registry. We were unable to analyze if drug persistence was a risk factor for occurrence of thromboembolic outcomes, because the follow-up was too short to allow statistical inference. Low adherence to anticoagulant prescription is potentially associated with increased risk of thromboembolism, but is notoriously difficult to capture and was not assessed in our investigation. There was no independent adjudication of study outcomes, but we validated outcomes internally according to criteria stated in the methods section. However, the data quality was ensured by performing all patient interviews in person with overall very good patient compliance to study procedures and a low dropout rate (4.8%).

## Conclusion

Patients with AF in this hospital setting had increased risk of thromboembolism and bleeding, which was mediated by the presence of comorbid conditions, especially a history of prior cardiovascular or cerebrovascular diseases. Further, anticoagulation treatment was frequently discontinued due to bleeding or thromboembolic complications, but also due to reasons such as permanent return to sinus rhythm, patient opinion or physician’s advice. The risk of thromboembolism in the group of hospital-patients should not be underestimated and patients should be encouraged to utilize follow-up visits specifically concerning anticoagulation treatment in the first year after anticoagulation initiation.

## Abbreviations

AF: Atrial fibrillation
CI: 95% confidence interval
CRNM bleeding: Clinically-relevant non-major bleeding
DOACs: Direct oral anticoagulants
DVT: Deep vein thrombosis
ECG: Electrocardiogram
eGFR: Estimated glomerular filtration rate
HR: Hazard ratio
IDMS: Isotope-dilution mass spectrometry
INR: International normalized ratio
LMWH: Low-molecular-weight heparin
MDRD: Modification of diet in renal disease equation
PE: Pulmonary embolism
TIA: Transient ischemic attack
VKA: Vitamin-K-antagonist
VTE: Venous thromboembolism
